# Pleuro-Pneumonia

**Published:** 1857-09

**Authors:** J. T. Jamieson

**Affiliations:** South Otselic, Chenango Co. N. Y.


ART. II. — *Pleuro-Pneumonia. Bleeding in the Recumbent Position.* By J. T. JAMIESON, M. D., South Otselic, Chenango Co., N. Y.

"Under no circumstances ought a patient to be blooded whilst his head is nearly on the same level with the trunk." — *Copland's Med. Dict. p.* 225.

We have here, Mr. Editor, one of the most learned and eminent men in our profession, inculcating a positive rule from which there is to be no exception; and if the doctor is correct in his assertion, I certainly violated the rule in the following case, and my practice must be considered as bad indeed. The case occurred several years ago, and the patient being a near neighbor, I had the opportunity of visiting him frequently.

L. Cook, æt. 51, a farmer, and possessing a somewhat nervous and excitable temperament, as indicated, in part, by a slightly tremulous state of the hands. I was informed that about twenty years ago he had inflammation of the lungs or pleura, or both, for which he was bled several times, and so reduced, that he did not perfectly recover his strength for about twelve months. Since that period, he has generally enjoyed good health. For the last three weeks, he has been laboring under a severe cold, but not so as to confine him to the house.

Feb. 21st. He was seized with severe rigors, and felt miserably.

22d. He had a thorough sweat, and afterward took a dose of oil. On the afternoon of this day, I saw him for the first time. He was perspiring moderately; tongue coated, and disposed to be dry in the centre; pulse a little accelerated; respiration but little affected; has some cough; complains of a pain, or rather soreness, low down on the right side, and feels the pain when he coughs, but not severely, and can lie down on either side.

Mustard poultice to the side; pil. hydr. and pot. bi-tart.

23d. Mane. Appears better; less pain in the side; tongue not so dry, and fever less; has pain in the head, especially on the right side.

Cont. Med.

Vespere. Worse in every respect. Fever high; pain in the head distressing; pulse 93, full, and bounding; pain in the side not much worse; cough dry, and expectoration difficult; sputa present a decided rusty tinge; respiration a little accelerated.

I immediately opened a vein in the arm, having a teacup in the basin. By the time the teacup was full, he fainted away, and fell back on the bed. Having recovered in a few moments, he was again placed in the sitting position, and about two-thirds of a teacupful more flowed into the basin, when he became generally convulsed, and fell back on the bed again. He recovered immediately. The arm was now bound up, and the patient expressed himself as relieved. I saw him again in a couple of hours, and found that the pulse had regained nearly the same full and bounding character which it had presented before the bleeding; skin hot, but moist. The blood in the teacup much buffed and cupped, that in the basin not at all, but forming a solid mass of coagulam with only a few drops of serum. There was a deep conviction on my mind, that the patient ought to lose more blood, and that the vis a tergo., imparting such a character to the pulse, must be subdued. A vein was therefore opened in the other arm, having a teacup in the basin as before. The blood flowed freely, and before the teacup was full, he was again convulsed, and fell back on the bed. He recovered immediately, and having the impression that he ought to lose more blood, I allowed it to flow to about twenty ounces, whilst he lay in the recumbent position. He appeared to bear it well, and represented himself as much relieved. Pulse soft and small.

Pil. hydr., and pulv. Doveri, every four hours, and small doses of ant. tart. every two hours.

24th. Mane. Decidedly better. Pulse 100, small and soft; fever less; has not coughed much, and expectoration more free; still feels some soreness in the side; tongue coated in the middle, and clean at the edges; skin moist during the night; bowels moved last evening, the evacuation being thin, and of a dark green color; blood both in teacup and basin cupped and buffed, thet in the teacup being the most so.

Cont. pil. et ant. tart.

1, P. M. Patient not so well; fever increased; pulse regained its full and bounding character; pain of the side increased since morning, and appears to the patient as if extending upward. A vein was opened for the third time, the patient being placed in the sitting position, but he became faint immediately, and being laid back on the bed, about sixteen ounces of blood was removed into a teacup and basin as before. He was considerably affected by the bleeding, and immediately after the arm was bound up, became as before generally convulsed. He recovered in a few moments, a few drops of spt. æth. nitros. being then given to him.

Calomel and pulv. Doveri every two hours. Solut. sod. bi-carb. p. r. n. empl. lytta lateri.

Visited patient again in about two hours, and found him improved. Pulse soft and small; skin quite moist, and he has been sweating considerably since the bleeding; blood, both in teacup and basin, as much buffed apparently, as was that last abstracted, but not so much cupped; serum of a yellowish green color.

Cont. Med.

9, P. M. Still improving. Pulse 94, soft and small; skin quite moist, and he has kept perspiring since last visit; respiration easy; tongue moist and much cleaner; blister rising.

Cont. Med.

25th. Mane. Appears to have a little more fever this morning; pulse soft, but rather fuller than last evening; respiration natural; expectorates more freely, but the sputa still exhibit the rusty tinge. Had rather a restless night, owing, in some measure, to the blister, which pained him severely. Skin moist; tongue rather more coated; bowels moved once, the evacuation consisting of dark colored slimy matter.

Cont. pil. every three hours.

Vespere. A little more feverish and restless; pulse small and soft.

Cont. pil.

26th. Better. Pulse 94, and soft; tongue cleaning; expectoration rather difficult.

Ol. ricini cochl. mag., and after operation, continue pills.

Vespere. Oil not operated. Pulse 93.

Rep. oil.

27th. Mane. Bowels moved several times since last evening, the evacuations being of a dark green color. Pulse 72 and soft; tongue cleaning; coughs but little, and expectorates easily.

Omit pills, and give a small dose of pulv. rhei and magnes. calcin.

Vespere. Bowels moved several times during the day, the evacuations being much the same, with the exception of the last, which consisted of about a tablespoonful of jelly-like matter, tinged with blood; pulse 70; tongue cleaner; countenance improved.

Powders of pulv. Doveri and hydr. c. creta.

28th. Pulse 62; has had a good night; bowels not moved; has taken two of the powders.

From this date the patient rapidly convalesced, and was soon about his business, having enjoyed good health up to the present time.

*Remarks.* I regard the above patient as being the subject of a peculiar idiosyncrasy in relation to the loss of blood. I admit that this term is only a cloak to our ignorance, but it serves very conveniently to convey the idea of a particular condition of the system, concerning the essential nature of which we know nothing. I suppose the idiosyncrasy, in the present case, to have had its seat more particularly in the brain. It is obvious that the fainting and convulsions manifested by the patient, a hard working farmer and the subject of so acute and violent an inflammation, during the two first bleedings, and when only a few ounces of blood had been removed, could not be ascribed to the absolute loss of blood, but must, it appears to me, be attributed to a particular and inexplicable state of the nervous system. The practical point, however, is, and which has been my inducement to forward the case for publication, if it be, like the law of the Medes and Persians, unchangeable and without exception, that "under no circumstances ought a patient to be blooded whilst his head is nearly on the same level with the trunk," what method of treatment would have been applicable to the above case? That the loss of blood was imperatively demanded can, I think, be questioned only by Botanics and all that genus, and under the circumstances, it only remained to resort to local depletion, the means for which were not at command; and supposing they had been, it does not seem to me that the local abstraction of blood would have availed anything, unless carried to the extent of influencing the general circulation, diminishing the vis a tergo, and subduing that full and bounding pulse, the instant feel of which, by the experienced physician, in an uncomplicated case of acute inflammation of the lungs and pleura, conveys to his mind the strongest conviction that the patient ought to lose blood, and that freely. Such being the case, I am unable to perceive any essential difference between abstracting a certain quantity of blood by leeches or cupping, the patient being in the recumbent position, and the removal of a like quantity from a vein whilst in the same position. In the local abstraction of blood, as by leeches, the patient being recumbent, we have frequently to judge of the effect of such loss of blood, and the extent to which it ought to be carried, by the aspect of the patient's countenance, and the state of the pulse, and why not be able to avail ourselves of the same means where it should seem necessary to resort to general depletion in the recumbent position? There can be no question that the rule inculcated by Dr. Copland, ought to be generally observed, and that the occasions for deviating from it will be rare, as also that much care and judgment ought to be exercised whenever such deviation is made; but to affirm that under *no circumstances* it ought to be departed from appears to me like laying it down too absolutely, and that the preceding case affords an illustration of the same. Others may think just as they please, but I am under the fullest conviction that had no more blood been abstracted from my patient than could have been obtained whilst he remained in the stting position, instead of being, as now, in the possession of good health, and managing his farm of fifty acres, he would, most probably, have been mouldering in the narrow house appointed for all living. I willingly admit that the third bleeding was hazardous, but yet regard it as proper under the circumstances, and as constituting the turning point in the case. It is not improbable, however, that could leeches have been applied to the side after the second bleeding, followed by a blister, or even the blister per se, the third bleeding might have been unnecessary, and such would be the practice I should adopt under like circumstances, in any future case, giving calomel also more early.

